# Host defense peptides combined with MTA extract increase the repair in dental pulp cells: in vitro and ex vivo study

**DOI:** 10.1038/s41598-023-36748-3

**Published:** 2023-06-12

**Authors:** Poliana Amanda Oliveira Silva, Danilo César Mota Martins, Ana Paula de Castro Cantuária, Rosangela V. de Andrade, Cristiano Lacorte, Jeeser Alves de Almeida, Lana Ribeiro Aguiar, José Raimundo Corrêa, Ingrid Gracielle Martins da Silva, Octávio Luiz Franco, Taia Maria Berto Rezende

**Affiliations:** 1 Programa de Pós-Graduação em Ciências da Saúde, Universidade de Brasília, Brasília, Distrito Federal, Brazil; 2 Programa de Pós-Graduação em Ciências Genômicas e Biotecnologia, Universidade Católica de Brasília, SGAN 916N – Av. W5 – Campus II – Modulo C, Room C-22170.790-160, Brasília, Distrito Federal, Brazil; 3 Laboratório de Biologia Sintética, Embrapa Recursos Genéticos e Biotecnologia, Brasília, Distrito Federal, Brazil; 4 Curso de Educação Física, Universidade Federal de Mato Grosso do Sul, UFMS, Campo Grande, Mato Grosso do Sul, Brazil; 5 Laboratório de Microscopia e Microanálises, Instituto de Ciências Biológicas, Universidade de Brasília, Brasília, Distrito Federal, Brazil; 6 S-Inova Biotech, Pós-Graduação em Biotecnologia, Universidade Católica Dom Bosco, Campo Grande, Mato Grosso do Sul, Brazil; 7 Curso de Odontologia, Universidade de Brasília, Brasília, Distrito Federal, Brazil

**Keywords:** Microscopy, Biological techniques, Cell biology, Materials science, Biomaterials

## Abstract

Host Defense Peptides (HDPs) have, in previous studies, been demonstrating antimicrobial, anti-inflammatory, and immunomodulatory capacity, important factors in the repair process. Knowing these characteristics, this article aims to evaluate the potential of HDPs IDR1018 and DJK-6 associated with MTA extract in the repair process of human pulp cells. Antibacterial activity of HDPs, MTA and HDPs combined with MTA in Streptococcus mutans planktonic bacteria and antibiofilm activity was evaluated. Cell toxicity was assayed with MTT and cell morphology was observed by scanning electron microscopy (SEM). Proliferation and migration of pulp cells were evaluated by trypan blue and wound healing assay. Inflammatory and mineralization related genes were evaluated by qPCR (IL-6, TNFRSF, DSPP, TGF-β). Alkaline phosphatase, phosphate quantification and alizarin red staining were also verified. The assays were performed in technical and biological triplicate (n = 9). Results were submitted for the calculation of the mean and standard deviation. Then, normality verification by Kolmogorov Smirnov test, analyzing one-way ANOVA. Analyses were considered at a 95% significance level, with a p-value < 0.05. Our study demonstrated that HDPs combined with MTA were able to reduce biofilms performed in 24 h and biofilm performed over 7 days *S. mutans* biofilm (p < 0.05). IDR1018 and MTA, as well as their combination, down-regulated IL-6 expression (p < 0.05). Tested materials were not cytotoxic to pulp cells. IDR1018 induced high cell proliferation and combined with MTA induced high cellular migration rates in 48 h (p < 0.05). Furthermore, the combination of IDR1018 and MTA also induced high expression levels of DSPP, ALP activity, and the production of calcification nodules. So, IDR-1018 and its combination with MTA could assist in pulp-dentine complex repair process in vitro.

## Introduction

Maintenance of pulp vitality is essential for preserving dental structure and function^1^. Through maintaining pulp vitality, it is possible to induce closure of the root apex, an important characteristic for root thickness and length development. This fact will result in fracture resistance and greater longevity of the tooth that had its pulp tissue exposed. One method for preserving pulp tissue after its exposure is performing a direct pulp capping^2^. Direct pulp capping is a procedure in which the material is placed directly onto the exposed pulp to preserve its vitality and promote repair. It is usually indicated for cases where the pulp exposure is accidental and limited, without signs of irreversible pulp inflammation or necrosis^2^. This technique can use biomaterials, such as calcium hydroxide and calcium silicate-based materials, for example: Mineral trioxide aggregate (MTA), Portland cement, Biodentine, BioAggregate, Endocem and others as cited by a previous publication^3^. All these afore mentioned repairing sealers have the aim of stimulating the healing and repair of lost tissue through reparative dentine production^4^.

The use of MTA is increasingly widespread in pulp capping practices^2^. Despite the satisfactory results with the use of this biomaterial, it has undergone developments over the years, with improvements in its manipulation, reduction in the size of its particles, and removal of components that caused dentin discoloration, such as bismuth oxide^5^. The appearance of the MTA Repair HP (Angelus) biomaterial is indicative of evolution. This biomaterial offers effortless manipulation thanks to the inclusion of a plasticizer and particle reduction. Moreover, it incorporates calcium tungstate as a radiopacifier, effectively reducing the potential for dentin pigmentation^6^. These new MTA formulations are still considered recent in the market and require more studies in comparison to other materials already established in endodontics.

For the repair process, characteristics such as antibacterial and immunomodulatory activities, in addition to the formation of mineralized tissue, are desirable for a pulp capper biomaterial^2,7,8^. Currently, calcium hydroxide, Biodentine and MTA formulations are the most used materials for pulp protection^1^. However, an overall success rate of 84% for MTA has been reported compared to calcium hydroxide (success rate of 59%) and 86% for Biodentine after 36 months^9^. This previous study demonstrated that the success of calcium hydroxide reduces with long-term follow up, while that of MTA and Biodentine remained reasonably stable^9^. The success of using existing biomaterials in therapy still depends on the duration of pulp exposure to the external environment, the size of the pulp exposure, patient’s age, and the degree of pulpal inflammation^10^. Therefore, there is still a search for new biomaterials that improve physical–mechanical properties and enhance biological properties such as antimicrobial and immunomodulatory activities^1,4,11–13^.

Thus, Host Defense Peptides (HDPs) have been investigated in the medical and dental fields due to their immunomodulatory, antimicrobial, antibiofilm, antiviral, antifungal, antiparasitic and chemotactic properties, among other applications^14^. HDPs actively participate in innate immunity; they also modulate the immune system's response to promote the clearance of pathogens while decreasing the deleterious effects of inflammation^15^. Likewise, they can also regulate adaptive immunity transition and promote wound healing^15^. Peptides can be classified as either natural or synthetic, based on their origin. As natural peptides have a short half-life, due to their rapid degradation caused by about 600 different proteases in the human body^16^, several strategies have been used to produce stabilized analogues, preserving the biological activities of the original molecules^17,18^. Different peptides have previously been tested. Among the natural peptides, there are studies related to the LL37 and Synoeca peptides. Among the synthetic peptides, there are studies related to Clavanin MO, IDR1002, IDR1018, DJK-6. Other areas of medicine have already demonstrated the ability of the IDR1018 peptide to eradicate *Staphylococcus epidermidis* biofilm, modulate the innate immune response, and promote osseointegration in orthopedic implants. Thus, there is a need to assesswhether the peptide would also promote antibacterial and repairing activity in factors corresponding to the oral cavity^19^.

Using the Bac2a sequence as a template, the origin of Host Defense Peptide IDR1018 resulted in a 12 amino acid sequencethrough point substitutions, shuffling, and deletions^20^. HDP IDR1018 has been reported in the literature as an agent with a wide range of biological activities, including pro- and anti-inflammatory functions^20^, minimal cytotoxic activities, and hemolytic activity, in addition to potent antibiofilm activity^21,22^. In 2015, a D-enantiomer version of IDR1018 was created from observing essential characteristics derived from this peptide and from the most active anti-biofilm peptides described in a previous study, giving rise to HDP DJK-6^23^. The peptide DJK-6 has a sequence of 12 amino acid residues (VQWRRIRVWVIR), with a molecular weight of 1667.62 Da. It was created to overcome the limitations of L-enantiomer peptides, which involve enzymatic degradation by host proteases. Thus, due to the D-amino acid version of DJK-6, this peptide tends to be more active and resistant to proteases^23^, reducing the possibility of enzymatic degradation by the host itself during possible therapies^22^.

Knowing the antibacterial and immunomodulatory characteristics of HDPs and  considering the potential limitations of MTA in these aspects, these peptides hold promise for enhancing such properties in this repair sealer. This fact is justified, since microbial contamination of the pulp dentine complex can affect repair, as well as the lack of control of pulp inflammation. In addition, the union of HDPs with other materials has already been evaluated previously, demonstrating improvement in several materials already tested^13,24,25^. Therefore, our study aimed to evaluate the potential of HDPs IDR1018, DJK-6, and their association with MTA extract, in the repair process of the pulp-dentine complex in a culture of human pulp cells. The null hypothesis was that the association of the peptides with the biomaterial MTA doesn´t contribute to an increase in the antibacterial, immunomodulatory and mineralization activities.

## Results

### Antimicrobial activity

**Figure 1:**
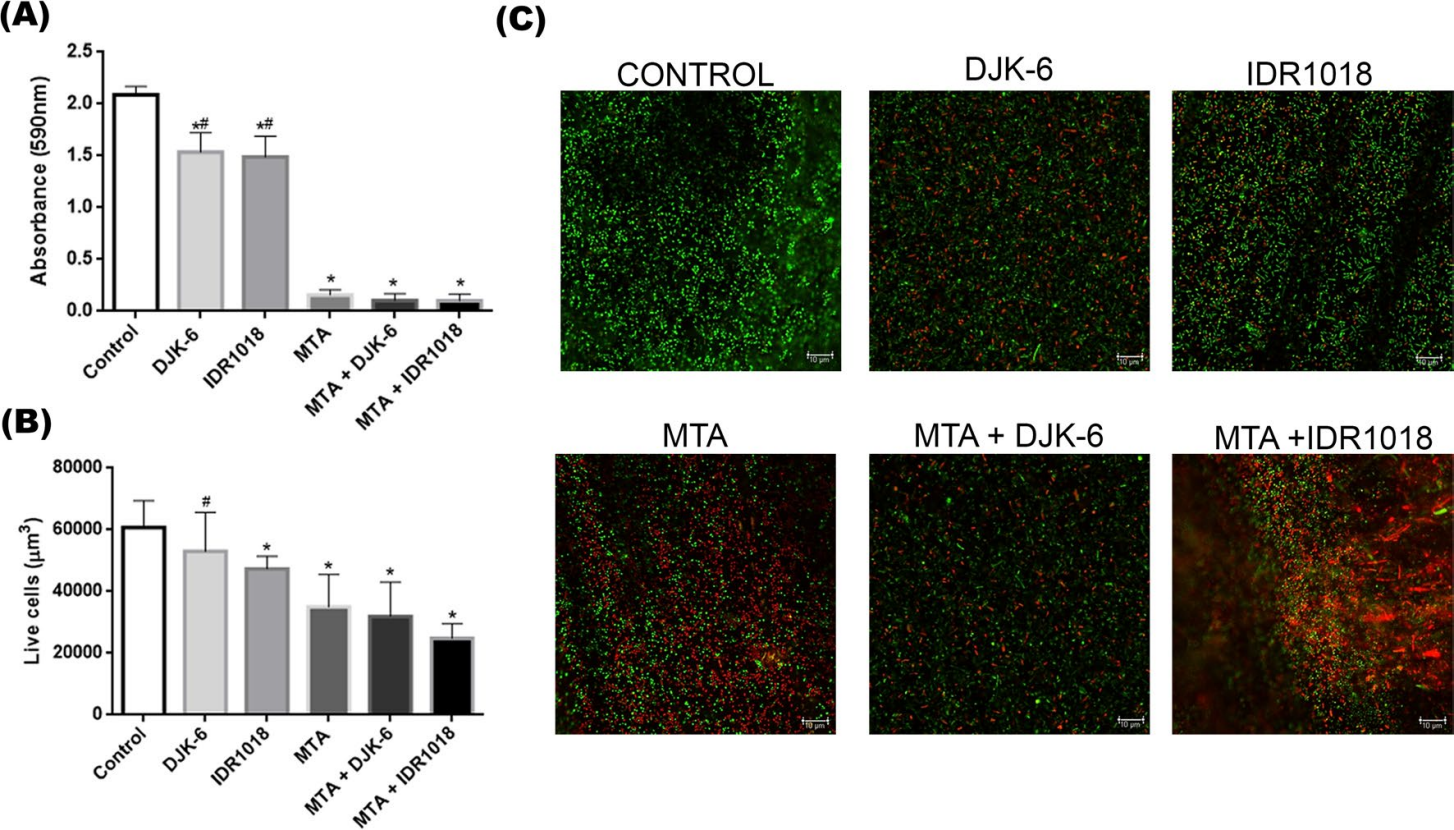
(**A**) *S. mutans* cell viability of *S. mutans* in a 24 h performed biofilm expoused to HDPs, MTA and combination of both. (**B**) Total biovolume (µm^3^) of living *S. mutans* in biofilm performed over 7 days on dentin slices followed by HDPs, MTA and combination of both exposure for 24 h, evaluated by confocal microscopy. Data were presented as mean ± standard deviation. *p < 0.001 in relation to control and #p < 0.001 in relation to MTA, by one-way ANOVA test and Tukey’s post-test. Control for (**A**) and (**B**) was represented by *S. mutans* bacterium in BHI medium. (**C**) Confocal microscopy images of *S. mutans* biofilm. Green images represent living cells and red images represent dead cells. Scale bar—10 μm.

After 24 h, neither MTA Repair HP extract, HDPs, nor the combination of HDPs and MTA were able to inhibit *S. mutans* growth under planktonic conditions in microdilution assay (data not shown). However, a significant reduction was noted in *S. mutans* biofilms performed in 24 h viability when cultures were exposed to MTA (92%), combinations of MTA with DJK-6 (95%), and IDR1018 (95%), when compared to non-stimulated cultures (p < 0.001) (Fig. 1A). Further, a 42% reduction in *S. mutans* viability in biofilm performed over 7 days biofilm was observed when the biofilm was exposed to MTA, 47% when exposed to MTA and DJK-6, and 59% when exposed to MTA and IDR1018 (p < 0.001) (Fig. 1B,C). Thus, MTA and HDPs in combination cause a reduction in the viability of *S. mutans* biofilm.

### Effect of HDPs, MTA, and a combination in an inflammatory environment

**Figure 2:**
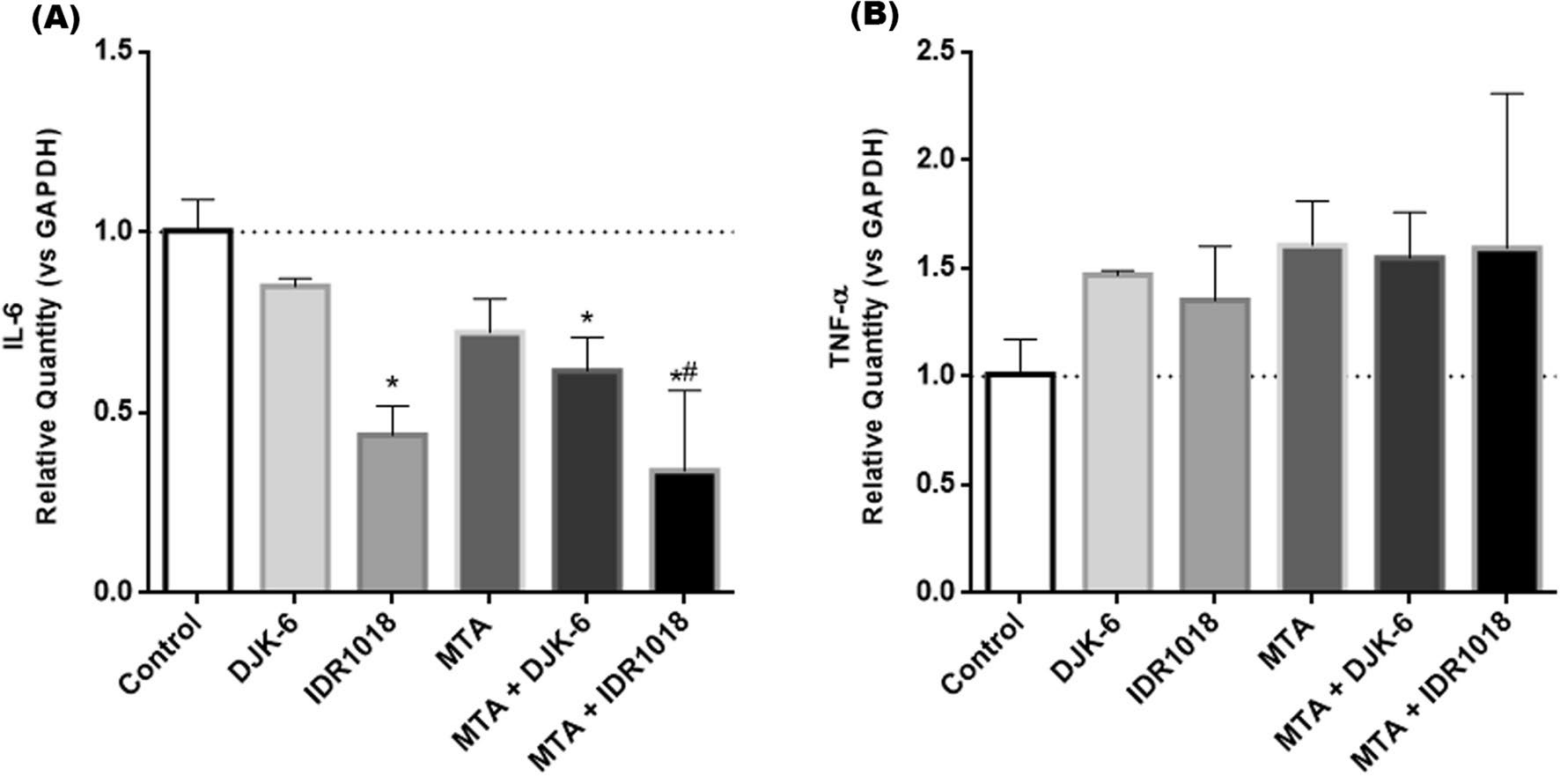
(**A**) Evaluation of IL-6 gene expression by qPCR, after 24 h exposure of HDPs, MTA and combination of both in pulp cell cultures in an inflammatory environment (LPS and IFN-γ—1 μg mL^−1^). (**B**). TNF-α gene expression after 24 h exposure to HDPs, MTA and combination of both in pulp cell cultures in an inflammatory environment. Control represented by cells stimulated by LPS and IFN-γ. Graphs represent the mean and standard deviation of the count of three independent replicates in technical triplicate. Statistical difference between groups verified by one-way ANOVA test and Tukey's post-test. *p < 0.05 in relation to control and #p < 0.05 in relation to MTA.

After microbial reduction, inflammatory control is an important factor during the repair process. Thus, the modulation of IL-6 gene expression in an inflammatory environment was evaluated in the presence of HDPs, MTA and combination of both. After 24 h, IDR1018, and a combination of HDPs and MTA down-regulated the IL-6 expression in an inflamed-pulp cell condition (p < 0.05) (Fig. 2A). It was observed that when MTA was combined with the IDR1018 peptide, there was a greater reduction in IL-6 compared to MTA alone, in an inflamed-pulp cell condition (p < 0.05) (Fig. 2A). However, no significant differences were observed in TNF-α expression when compared to the control (Fig. 2B).

### Cytotoxic and morphologic evaluation of pulp cells

**Figure 3:**
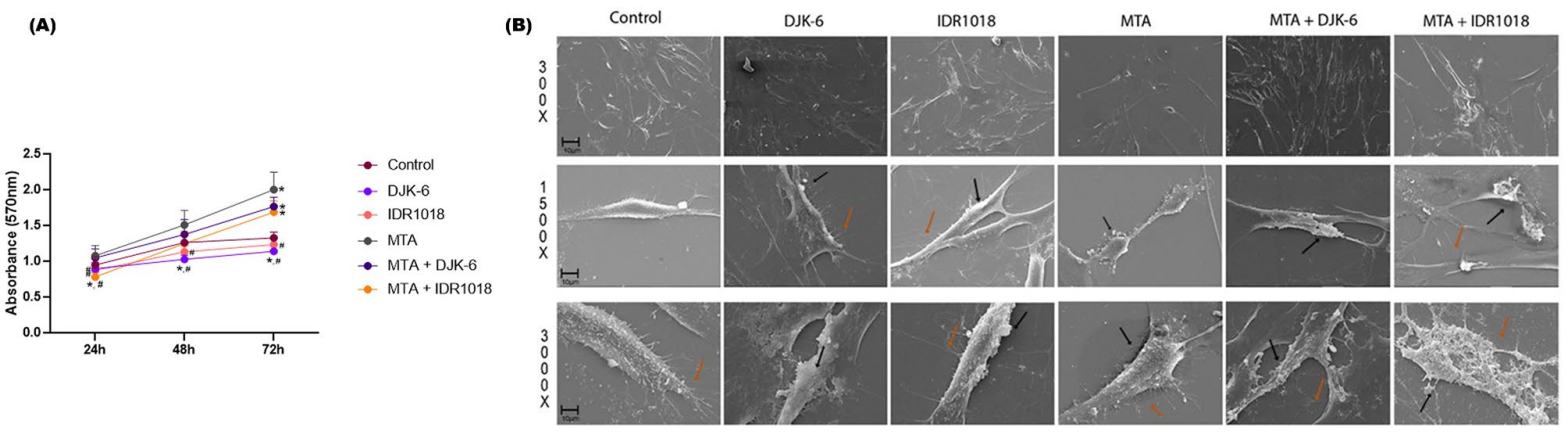
(**A**) Pulp cell viability by MTT, after exposure of HDPs (DJK-6 and IDR1018), MTA and combination of both after 24 h, 48 h and 72 h in pulp cell culture. Data were presented as mean ± standard deviation. *p < 0.05 in relation to control and #p < 0.05 in relation to MTA Repair HP. Statistical differences verified by the ANOVA one-way test and Tukey’s post-test. (**B**) Scanning electron microscopy representative of pulp cell culture after exposure to negative control (DMEM). HDPs, MTA biomaterial and combination of both were evaluated. Each line represents the increase used for sample analysis. Material tested for each analysis is represented in the columns and the lines represent the evaluated magnification (300×, 1500× and 3000×). Orange arrows demonstrate cytoplasmic extensions and black arrows represent aggregates of materials on the cell surface.

MTA repair HP extract, HDPs, and combination of DJK-6 and MTA were not toxic to pulp cell culture, after contact for 24 h, when compared to control. However, the MTA extracts containing the peptide IDR1018 promoted a reduction of 17% in cell viability when compared to the control (p < 0.05). After 48 h, DJK-6 promoted a cell viability reduction of 18.5% (p < 0.05). Nevertheless, IDR1018, as well as DJK-6 and IDR1018 combined with MTA, were capable of maintaining cell viability. Furthermore, after 48 h, no cytotoxicity of IDR1018 combined with MTA was observed when compared to the control. After 72 h, a reduction of 14% in pulp cell viability was caused by DJK-6 (p < 0.05) (Fig. 3A).

Scanning electron microscopy analyses, at 300 × magnification, presented elongated pulp cells in all tested conditions (Fig. 3B). However, there was a reduction in the number of cytoplasmic extensions in focal adhesion points in MTA-exposed cultures. It was also possible to observe a greater presence of thin cytoplasmic filaments adhered to the coverslip in control and DJK-6 and IDR1018 peptide-exposed cultures. However, a reduction in these cell filaments was observed in samples exposed to MTA and the combination of MTA and HDPs. The presence of aggregates was also noted in the cell membrane of cells exposed to HDPs and MTA (3000 × magnification). Besides, adding the peptides DJK-6 and IDR1018 to the MTA extract produced an even more abundant aggregation on the cell surface, mainly in IDR1018 peptide and MTA-exposed cultures (Fig. 3B).

### Pulp cell migration and proliferation

**Figure 4:**
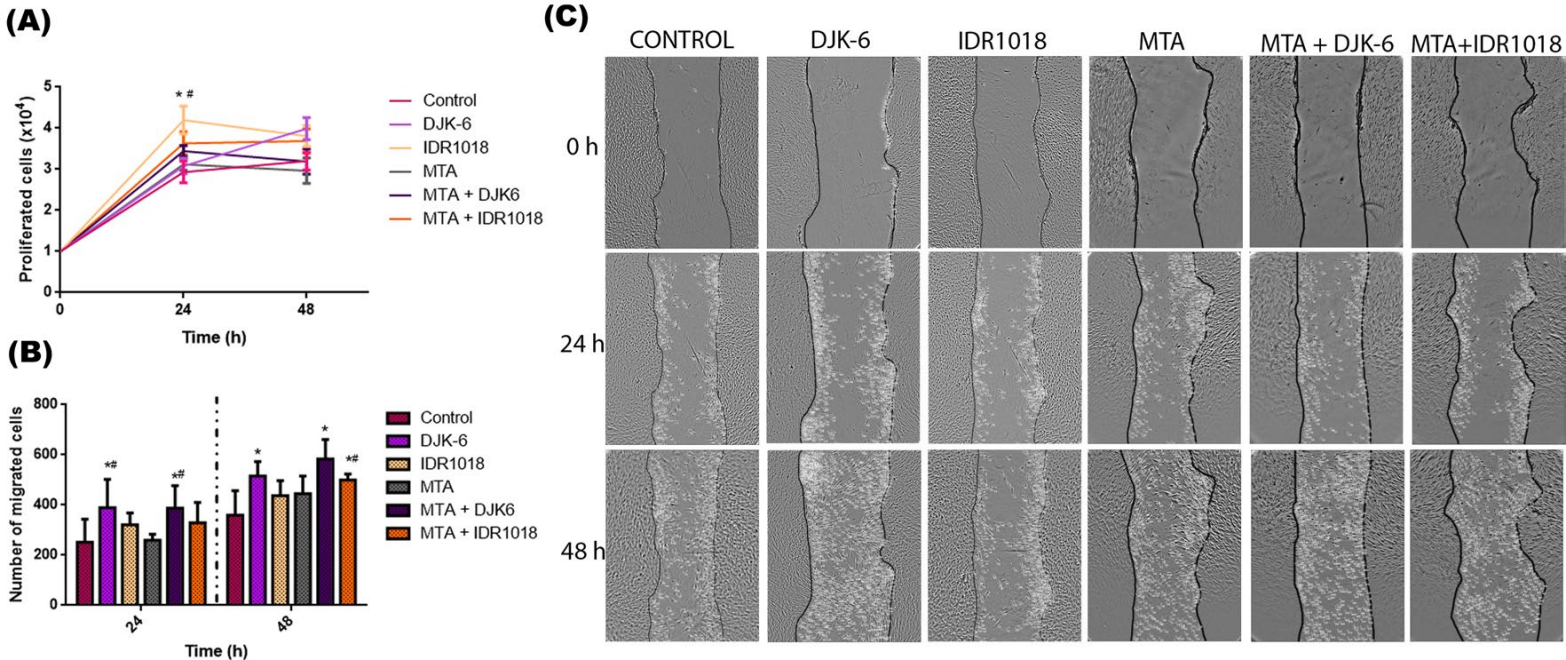
(**A**) Pulp cell proliferation by trypan blue exclusion method, 24 h and 48 h after exposure to HDPs, MTA and combination of both. Observing statistical difference only for the IDR1018 peptide in the 24 h period with the control and with MTA. (**B**) Pulp cells’ migratory behavior when exposed to HDPs, MTA and combination of both. Representative graph indicates number of cells that migrated into the wound at 24 h and 48 h. Data were presented as mean ± standard deviation. *p < 0.05 in relation to control and #p < 0.05 in relation to MTA Repair HP. Statistical differences verified by the ANOVA one-way test and Tukey’s post-test. (**C**) Representative image of the cell migration test, using the scratch method. Dots denote the presence of cells inside the wound. Vertical lines correspond to the initial place where the wound was performed at the starting point. The test was performed for 24 h and 48 h. Columns represented each tested material.

After 24 h of contact with HDPs, MTA repair HP and a combination of both in pulp cell culture, greater proliferation was observed when cells were exposed to IDR1018, when compared to control and MTA (p < 0.05) (Fig. 4A). To assess cell migration after exposure to HDPs, MTA and a combination of HDPs and MTA, a migration assay was performed. After 24 h, DJK-6 and the combination of MTA with DJK-6 increased cell migration by 55%, compared to the control (p < 0.05) (Fig. 4B,C). In addition to exposing the cells to the MTA and DJK-6 for 24 h, there was a 51% increase in cell migration, compared to MTA (p < 0.05). After cell exposure to HDP DJK-6 for 48 h, an increase in migration of 44% was observed, compared to the control (p < 0.05). After 48 h of exposure to DJK-6 and IDR1018 combined with MTA, increases of 62% and 39% were evaluated, respectively, compared to the control (p < 0.05) (Fig. 4B,C). Thus, IDR1018 probably promotes pulp cell proliferation, while peptide DJK-6 alone or combined with MTA leads to pulp cell migration.

### Odontogenic differentiation and biomineralization

**Figure 5:**
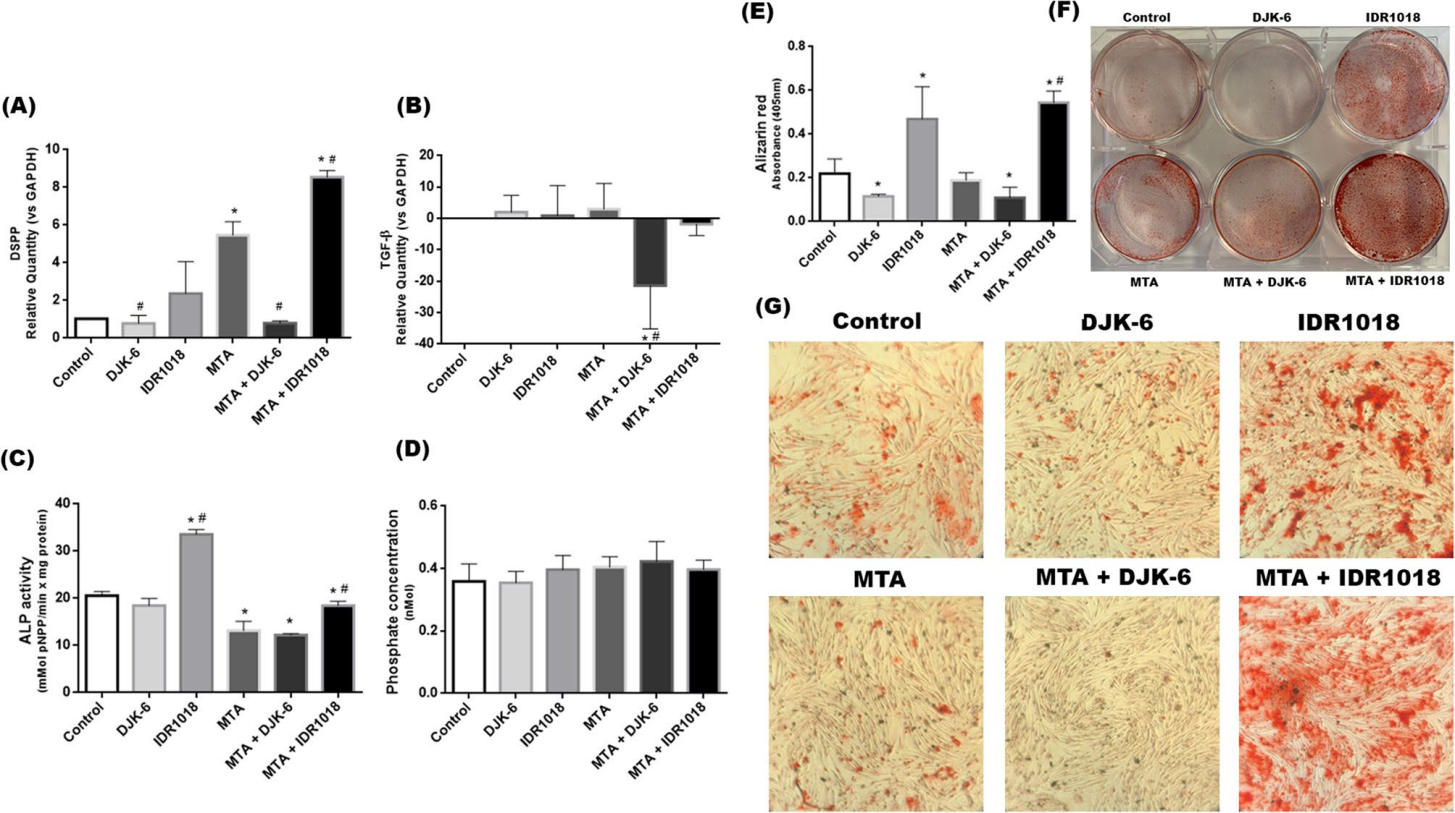
(**A**,**B**) Pulp cell expression of DSPP and TGF-β by qPCR, after 14 days of exposure to MTA, HDPs and combination of both in osteogenic medium. (**C**) Pulp cell alkaline phosphatase (ALP) activity in osteogenic conditions, for 14 days. ALP activity (mMol p-nitrophenol released per min) was normalized by total amount of protein. Graphical representation of three independent biological replicas. (**D**) Evaluation of the phosphate concentration of the cell supernatant, after exposure HDPs, MTA and combination of both for 14 days. (**E**) Pulp cell mineral matrix deposition after 14 days by alizarin red staining. Data were presented as the mean ± standard deviation percentage of staining compared with the control (*p < 0.05 in relation to control and #p < 0.05 in relation to MTA Repair HP, by one-way ANOVA and Tukey’s post-test). (**F**) Representative image of mineralized matrix in 6-well plate after 14 days of exposure to HDPs, MTA and combination of both (**G**) Representative image of mineralized matrix deposition by alizarin red staining in odontogenic differentiation conditions and espoused to HDPs, MTA and combination of both. Control represented pulp cell culture in odontogenic medium for 14 days.

MTA combined with IDR1018 improved DSPP gene expression in pulp cells, compared to non-stimulated cultures and MTA-stimulated cultures (p < 0.05). Meanwhile, DJK-6 and the combination of MTA and DJK-6 caused a reduction in DSPP expression, compared to cells exposed only to MTA (Fig. 5A) (p < 0.05). The combination of DJK-6 and MTA caused a reduction in TGF-β gene expression (p < 0.05) (Fig. 5B). MTA alone or combined with DJK6 caused lower ALP activity, compared to the control (p < 0.05). HDP IDR1018 and its combination with MTA caused higher ALP activity compared to control and MTA alone (p < 0.05) (Fig. 5C). However, no significant differences were observed between HDPs, MTA, and the combination of HDPs, and MTA with control regarding phosphate production (Fig. 5D). Concerning alizarin red assay, it was observed that HDP IDR1018 and its combination with MTA promoted greater production of calcification nodules, compared to control and MTA alone (p < 0.05) (Fig. 5E,F,G). However, it was observed that peptide DJK-6 alone or with MTA caused lower production of mineralized nodules compared to the control (p < 0.05).

## Discussion

Pulp-dentine complex regeneration and repair involve the balance between pathogen control and pulp inflammatory response^26^. These processes are followed by pulp cell migration and proliferation, mesenchymal stem cells differentiation, and the biomineralization process. Currently, as repair-inducing materials, calcium hydroxide and MTA formulations are available^1^. However, studies have shown that calcium hydroxide can promote a fragile and disorganized dentine structure formation compared to MTA biomaterial^2,27,28^. Thus, the use of MTA is increasingly widespread in pulp capping practices^2,29^. The success of endodontic conservative procedures depends on the control of microorganisms’ progression and the biocompatibility of repair-inducing materials^10^. Also, the material must have bioactivity. Taking this capability into account, pulp immune response modulation helps repair the pulp-dentine complex^10,12^. Observing the limitations of the currently used materials, in relation to repair time, tissue characteristics, and defects in mineralization nodules (Cox et al. 1996,Goldberg et al. 2004,Mente et al. 2014), HDPs were investigated here as possible adjuvants in this repair process, given their immunomodulatory, antibiofilm, and repair inducer potential. Therefore, the use of HDPs combined with MTA was considered, given their wide clinical use.

Previous studies have not shown any antibacterial effect of MTA on *S. mutans* under planktonic conditions^30–32^. Our findings demonstrated that although HDPs, MTA or a combination of both did not cause inhibition of *S. mutans* growth under planktonic conditions, these materials have the potential of reducing biofilm performed in 24 h and biofilm performed over 7 days biofilms of *S. mutans*. Our group also recently demonstrated the ability of MTA extracts to reduce biofilm performed in 24 h and *S. mutans* biofilm performed over 7 days^32^. Besides, previous studies have demonstrated that the antibiofilm capability of IDR1018 is directly related to its concentration^33,34^. It was also demonstrated the ability of high concentrations of HDPs to be more effective against biofilm performed in 24 h *S. mutans* biofilm. In this context, it was possible to observe that higher tested concentrations of the HDPs DJK-6 and IDR1018 increased the eradication of the MTA antibiofilm. However, it was noticed that with an increased concentration of peptides, there was also a reduction in pulp cell viability. Thus, knowing that the repair process corresponds to a set of events, we selected 16 µg mL^−1^ of each peptide concentration. Because this concentration was not toxic to the pulp cells and still promoted remarkable cell migration. Highlighting the need for standardization of peptide concentration reaching the pulp cells and the reduction of peptide degradation risk. Peptide was not manipulated together with MTA but rather added to the extract. As a future perspective, more studies are needed to analyze the best way to incorporate the peptides into a product with MTA, aiming to maintain the ideal concentration of both MTA and peptide for pulpal tissue interaction.

HDPs in association with other molecules have already been observed in previous studies, resulting in a strong reduction in *S. mutans* biofilm when peptide IDR1018 was associated with the peptide ZXR-2.3,which is considered a potent peptide with antimicrobial activities, derived from the toxin mauriporin of the Moroccan scorpion *Androctonus mauritanicus*^33^, or associated with chlorhexidine and EDTA, against oral multispecies biofilms^35^. The peptides’ ability to act effectively on biofilms, despite having little or no effect on planktonic bacteria, can be justified by the IDR1018 and DJK-6 antibiofilm mechanism of action. It seems that these peptides can bind to guanosine pentaphosphate [(p) ppGpp], which is an essential nucleotide present in biofilm formation and maintenance^20,36^.

The presence of microorganisms in the pulp-dentine complex activates a chain of inflammatory signaling^26,37^, including the release of TNF-α, a marker of early inflammation^38^. TNF-α promotes leukocyte recruitment, induces vasodilation, and stimulates the production of pro-inflammatory cytokines, including IL-6^39^. Here, no difference was observed in TNFRSF-1 gene expression after cell exposure to HDPs. It is important to mention that all extracts were tested in LPS and IFN-γ stimulated culture for 24 h, characterizing the inflammatory phase of the repair process. A previous study also showed an increase in TNF-α production when exposing macrophages to MTA in an inflamed environment^40^. In addition, MTA has the ability to increase TNF-α production in inflammatory stimuli-free pulp cell cultures^41^. However, our results also demonstrated a decrease in IL-6 gene expression after cellular exposure to HDP IDR1018, MTA, and the combination of both, in this immune-inflammatory environment. A similar reduction in IL-6 production was demonstrated in IDR1018-stimulated macrophage cultures^42,43^. Another study demonstrated the ability of MTA and the further combination of MTA and N-acetyl cysteine to decrease IL-6 and TNF-α in an inflamed pulp cell culture stimulated with LPS^44^, corroborating our findings. With the reduction of IL-6 and other inflammatory mediators, there will probably be a reduction of TNF-α, leading to a drop in its production but not zero production, given that the presence of TNF-α is still necessary to promote tissue repair^45^.

Another critical point for the pulp capping material, besides its antibacterial activity, is biocompatibility. It wasnoted that HDPs and MTA did not cause cytotoxicity in pulp cell cultures, as demonstrated in previous studies^12,46,47^. Only the combination of MTA and IDR1018 led to a reduction of 17% in cellular viability after 24 h, which was reversed after 48 h of incubation. A reduction of 18.5 and 14% in cell viability was also observed in HDP DJK-6-stimulated cells after 48 h and 72 h, respectively. These values are acceptable because they are lower than 30% of cell death, according to ISO 10,993–5-2009 (International Organization for Standardization, 2009). Materials such as Dycal (Dentsply, Johnson City, TN, USA) are widely used for indirect pulp capping. However, the literature demonstrates a 65% reduction in in vitro cell viability after 8 days of exposure^48^. Given the limitations of the MTT method, both the MTT method and the trypan blue method were used, which confirmed cell viability and cell proliferation.

In the next step, cell morphology was evaluated by scanning electron microscopy. In this assay, the aggregates were observed on the cell surface formed after HDPs, and the MTA biomaterial itself had been added. Just as with HDP IDR1018, it was observed aggregates on the cell surface after the addition of peptide DJK-6 and MTA biomaterial. Since peptide DJK-6 is derived from HDP IDR1018^23^, both might display similar properties. Previous reports also observed these precipitates on the cell surface after contact with MTA. However, there are no reports of decreased effectiveness or impairment depending on this characteristic^49–51^.

In our study, it was possible to assert that after the combination of HDPs with MTA, the presence of aggregates was more abundant, possibly caused by the somatic presence of precipitates of both materials. Furthermore, the presence of cell surface materials may have reduced the cytoplasmic elongations that cause adhesion to the glass. The presence of these precipitates on the cell surface may represent a direct interaction between MTA and cell culture since there were no significant reductions in cell viability, and still observed higher cell proliferation. This was observed when MTA combined with IDR1018-stimulated cultures were compared to MTA-stimulated cultures and control. Even though peptide DJK-6 caused a slight reduction in cell viability, it was noticed that the same peptide led to a higher migration rate in 24 h and 48 h. This, in turn, led to an increased in cell migration when DJK-6 was combined with MTA extract, compared to MTA-stimulated cells. The same relationship was observed when IDR1018 was added to MTA-stimulated cells. Previous data demonstrated that MTA promotes in vitro pulp cell migration leading to similar result as pulp cells control without any stimulation in the first 24 h and increasing wound closure in 48 h^12^. This corroborates our results and indicates that the combination of peptides with MTA may favor the migratory process in pulp cell cultures. A previous study demonstrates that IDR1018 causes cell proliferation and migration in 2D and 3D cultures of fibroblasts, keratinocytes, and melanocytes^52^. At lower concentrations (2 µg mL^−1^), IDR1018 promoted wound closure in mice^53^.

Through previous studies, migration characteristics are noted different when different MTA-based sealers produced by different companies were used^13,54,55^. For example, research with MTA Pro Root (Dentsply) has shown that the lower the dilution of the MTA extract, the better its migratory capacity^56^. Another difficulty observed in studies using MTA extracts is the current standardization of this extract, which only imposes temperature and contact time of the material with the conditioned medium. Thus, the concentration of the material that will come into contact with the cells and the final volume is variable in a variety of evaluated studies, making the standard of comparison with this material difficult.

In dentine-pulp repair, after inflammation control, cell migration, and proliferation, the dentine biomineralization process begins^26^. During this process, a protein of considerable importance is DSPP, which has been used as a marker for the in vitro differentiation of pulp-derived stem cells into odontoblasts^57,58^. Here, was observed that MTA and the combination of MTA with IDR1018 caused an increase in DSPP production, demonstrating the ability of IDR1018 to assist and improve the odontoblastic differentiation process and dentine mineralization. A previous study demonstrated that the addition of osteostatin protein to MTA increased the DSPP gene expression in pulp cell cultures^13^. Our study also observed low DSPP gene expression when HDP DJK-6 was added to pulp cell culture, as well as inhibition of TGF-β expression. TGF-β acts on the cell by regulating growth, differentiation, and extracellular matrix deposition^26,59,60^. Previous studies reported that TGF-β has a crucial role in regulating DSPP transcription^59,60^, which may explain the low DSPP expression when pulp cells were stimulated with HDP DJK-6. However, our study observed low TGF-β expression in the other groups and high DSPP gene expression. This contrasted with results shown by Hwang et al. (2008) and Niwa et al. (2018), which demonstrated that high concentrations of TGF-β decreased in vitro DSPP expression^61,62^. Moreover, a study performed in mice showed TGF-β overexpression caused by low DSPP expression, which compromised the dentine mineralization of mice^63^. Thus, TGF-β may be a critical factor in the transition from the non-mineralized predentine matrix to the mineralized matrix^61^. TGF-β expression may be high during the first stages of repair and after the mineral matrix deposition causes a reduction in its expression^61^.

Following the odontoblastic differentiation and mineral deposition, the enzyme alkaline phosphatase (ALP) is an early marker of calcification^64^. Here, it was showed that the peptide IDR1018 and the combination of MTA and HDP IDR1018 promoted a higher ALP activity. This fact demonstrates the ability of both molecules to promote a significant formation of mineralization nodules in comparison to the same formation in an osteogenic medium (control). Then, calcification nodules were observed when MTA Repair HP was used, similarly to the used control. Phosphate is an essential structural component in the formation of hydroxyapatite, and in our study, neither HDPs nor MTA led to a phosphate deposition reduction. The biomineralization process ends with the mineral matrix formation, and the addition of HDP IDR1018 combined with MTA increased the deposition of this tissue. This is an extremely important factor for the success of conservative endodontic therapies, given the need to produce dentine mineralization nodules^10^.

It is recognized that the study had some limitations, among them was the cultivation of monocultures of S. mutans. Monoculture cultivation does not fully reflect the complex interactions observed in multispecies biofilms within the oral cavity^65^. However, the use of monoculture in our investigation served as an initial exploration, allowing us to gain valuable insights into the antimicrobial activity of our formulations against this specific bacterium. The benefits of this approach include better control over experimental conditions, increased reproducibility, and the ability to isolate the effects of the investigated peptides and MTA. However, the importance of future studies that address the limitations of monoculture and incorporate multispecies biofilms is acknowledged. By including multiple species, a more accurate simulation of the clinical environment can be achieved, allowing for a better understanding of the formulations' antimicrobial efficacy in the context of diverse oral pathogens and their intricate interactions. Furthermore, in vitro studies have inherent limitations, including the simplified and controlled environment that does not fully represent the complexity of in vivo systems. The absence of tissue architecture and physiological conditions may limit the relevance of the findings. Despite these limitations, in vitro studies play a crucial role in elucidating fundamental mechanisms, testing hypotheses, and providing initial insights into the effects of interventions. Guiding subsequent in vivo and clinical investigations. Therefore, as a part of our future research plans, the group intend to culture and evaluate the peptides and MTA in the presence of multispecies biofilms, aiming to provide a more comprehensive assessment of their antimicrobial potential.

This study presents the first evaluation of DJK-6 action against planktonic bacteria and *S. mutans* biofilms. In addition to low cytotoxicity in pulp cell culture, it was observed that when DJK-6 was used in conjunction with MTA, a greater migration capability was observed. However, IDR1018 potency and its combination with MTA were able to control biofilm, and assist in the repair process, inducing cell proliferation and migration. The treatment with HDP and its combination with MTA participated in the biomineralization process, induced the expression of the DSPP gene, increased ALP activity, and caused larger mineralization nodules in vitro. Thus, formulations with HDP IDR1018 can help in the repair process of the pulp-dentine complex. Future in vivo trials using HDPs in combination with MTA can elucidate questions about the interaction of peptides with the complete system.

## Methods

### Human dental pulp cells isolation and culture

Cells from ten non-erupted open-apex teeth donated from patients aged 18 to 20 years, after signing the consent form, were used. Patients with third molars presenting carious lesions, fractures or periodontal disease were not invited to the study. After extraction, the teeth were placed in a Falcon tube containing 10 mL of Dulbecco’s Modified Eagle Medium—DMEM (Gibco®, Grand Island, NY, USA), with 200 µL of amphotericin B fungizone (Sigma Aldrich®, St. Louis, USA) and 10 µL of gentamicin (Sigma Aldrich®). The set was transported to the university laboratory and cultured within 2 h of extraction. Cells were cultured using the explant technique^66^. Dental pulp was removed and washed in phosphate-buffered saline (PBS). The pulp was sectioned with a scalpel blade and transferred to a 6-well plate in DMEM high glucose (25 mM) medium (Sigma Aldrich, St. Louis, MO, USA), 50 U mL^−1^ penicillin (Gibco, Grand Island, NY, USA), 50 µg mL^−1^ streptomycin (Gibco) and 20% fetal bovine serum (Gibco). Confluent cells were subcultured in DMEM medium supplemented with antibiotics and 10% fetal bovine serum. Cell cultures between the 3^rd^ and 6^th^ passages were used for cell viability experiments, cell morphology, migration, and proliferation. Pulp cells between the 3^rd^ and 4^th^ passage were used for odontogenic differentiation assays^67^.

### Preparation of MTA extracts

MTA Repair HP (Angelus, Paraná, Brazil) was prepared according to the manufacturer's instructions and used in all experiments as MTA extracts. Each MTA Repair HP (Angelus) containing 0.085 g was divided into four parts. After each part had been manipulated, it was inserted into a well of a 24-well plate (16.2 mm in diameter and 2 mm in height) and incubated at 37 °C, for 30 min for material setting^68^. After setting time (30 min), 2.5 mL of the standard used medium for each well was added. Plates were incubated at 37ºC, for 24 h, according to the International Organization for Standardization (ISO) 10,993–5. After that, the supernatant from the four wells were pooled and was filtered with sterile 0.22 mm filters^12,68^.

### HDP preparation

IDR1018 (VRLIVAVRIWRRNH2) and DJK-6 (VQWRRIRVWVIR) HDPs were synthesized by Peptide 2.0 (USA) with purity > 95%. Confirmation of molecular mass and purity was analyzed by MALDI-TOF mass spectrometry (Supplementary Fig. 1). Peptides were weighed and diluted in ultrapure Milli-Q water for each experiment. Peptides were added to MTA extract at the moment they were inserted in cell culture. Concentrations of HDPs on MTA extracts were defined based on the most significant cell migration data after 24 and 72 h, and which were non-cytotoxic to pulp cells. A concentration of 16 µg mL^−1^ of both peptides was selected to be added to MTA extracts and used in all experiments (Supplementary Figs. 2 and 3).

### Effect of HDPs, MTA, and combination of both on antimicrobial activity

The minimal inhibitory concentration of HDPs and combination of HDPs and MTA against *S. mutans* (ATCC 25175) activity against planktonic bacteria was determined according to the protocol previously proposed by Ji et al. (2011)^69^. The bacteria were cultivated under aerobic conditions, and after the growth curve, it was determined that an absorbance of 0.25 nm corresponded to 1 × 10^7^ CFU. The antibiofilm activity was performed in two situations: biofilms performed in 24 h in U-bottom plates, , and biofilms on dentine disk, performed over 7 days. The positive control was represented by the group containing antibiotic chloramphenicol (10 µg mL^−1^)^70^ and the negative control was represented by the well containing the culture of S. mutans in culture medium.

#### Biofilms performed in 24 h

Antibiofilm assays were performed with a pre-inoculum of *S. mutans* on Brain Heart Infusion medium (BHI medium) (Sigma) at 37ºC, 220 rpm, overnight. The bacterial suspension was diluted in modified basal medium 2 (BM2)—(62 mM potassium phosphate [VETEC, Rio de Janeiro, RJ,Brazil], 7 mM (NH_4_)_2_SO_4_ [VETEC], 2 Mm MgSO_4_ [Sigma‐Aldrich, St. Louis, MI], 10 μM FeSO_4_ [VETEC], and 0.5% glucose [Sigma-Aldrich])^71^ to 1/100 v/v per well and cultivated in a 96-well plate with U-bottom at 37 ºC, for 24 h. Preformed biofilm was exposed to HDPs, MTA repair HP (Angelus) extracts, and a combination of both. In order to assess the viability of the biofilms, a specific procedure was followed. Firstly, 100 µL of BM2 medium and 10 µL of 3-(4,5-dimethylthiazol-2-yl)-2,5-diphenyltetrazolium bromide (MTT) solution (Sigma) were carefully added to each well of the microplates. The microplates were then incubated in a dark environment at a temperature of 37ºC for a period of 4 h, as indicated by the experimental protocol^72^. Subsequently, once the cell product was completely solubilized, the absorbance of the samples was measured at a wavelength of 570 nm using a spectrophotometer^73^. This step allowed for the evaluation of the metabolic activity and viability of the biofilms. The test was performed in technical triplicate and three individual replicates, conducted on different days.

#### Biofilms performed over 7 days

To mimic clinical conditions, the biofilm was cultivated on dentin discs. So, ten caries-free crown third molars were used in the study. To prepare the specimens for analysis, the teeth crowns were carefully sectioned in the transverse direction using a diamond disc-cutting machine, ensuring that the cut was made precisely at the level of the pulp horn. Each tooth yielded a 1 mm thick and 4 mm diameter disc. The obtained discs were then subjected to a specific treatment protocol. Both sides of the discs were treated with 0.5 M EDTA solution for a duration of 60 s, followed by thorough rinsing with distilled water to remove any residual EDTA^74^. Subsequently, discs were individually wrapped in surgical-grade paper and sterilized in an autoclave (saturated steam under pressure).

Discs were inserted into the bottom of a 24-well plate and, sequentially, bacterial suspension of *S. mutans* (ATCC 25,175) was diluted in BHI medium to 10/990 v/v per well and cultivated under the disc at 37ºC, for 7 days. Next, the dentine disc and biofilm were removed from the well and gently washed with PBS, then inserted in a new 24-well plate. The biofilm was exposed to HDPs, MTA repair HP (Angelus) extracts, and a combination of both for 24 h. *S. mutans* bacterium in BHI medium was the control. After 24 h, the disc was washed in PBS twice to remove the culture medium and non-adherent cells. Then, the disc surface was stained with 50 µL live/dead baclight bacterial viability kit, composed of SYTO 9 and propidium iodide (Thermo Fisher Scientific, Waltham, MA, USA), SYTO 9 is a fluorescent dye that emits a green signal and can bind to both viable and non-viable microorganisms. On the other hand, propidium iodide is a red fluorescent dye that specifically enters cells with compromised membranes, indicating non-viability (dead microorganisms). Discs were incubated at room temperature for 10 min; rinsed with PBS and observed in a confocal laser scanning microscope (Leica TCS-SPE; Leica Biosystems CMS, Mannheim, Germany). A single trained evaluator, different from the person who performed the bacterial culture, selected 5 areas of the disk and standardized them for evaluation of all other disks. Then, five captures of each sample were made using a 40x/1.25 oil objective and a 488 nm laser. Each image was representative of a 275 × 275 µm^2^ field. Images were then transferred to the Imaris 7.2 software (Bitplane Inc, St Paul, MN). The parameters evaluated in each group were the total and green (live) biovolumes (mm^3^). The biofilm analysis tool was used to evaluate the 5 fields of each sample. The test was performed in two individual replicates, conducted on different days. The results for each group generated a single average, representative of 10 fields in each sample^73,75^. Afterwards, the data were presented in a graph, considering the live cells.

### Real-time polymerase chain reaction analysis

First, a cell system capable of simulating pulp cells' immune response was established. For this purpose, Interferon (IFN)-γ and lipopolysaccharide (LPS) were used to activate pulp cell response in culture. Effects of HDPs, MTA, and a combination of both were observed in all these tested conditions.

Thus, pulp cells were plated in technical triplicate in 6-well plates with supplemented DMEM medium, at a density of 2.5 × 10^5^ cell/well and stimulated with IFN-γ and LPS for 24 h. After that time, HDPs, MTA repair HP, and a combination of both were exposed to pulp cells for 24 h. Next, messenger RNA levels of interleukin (IL)-6 (Hs01011609_m1), tumor necrosis factor (TNF)-α (Hs00174128_m1), and GAPDH were evaluated with StepOnePlus™ Real-Time PCR System (Applied Biosystems, ThermoFisher Scientific, California, USA), under conditions recommended by the manufacturer. Total RNA was isolated from 1 mL of Total RNA was isolated from 1 mL of pulp cells' using the TRIzol Reagent® (Invitrogen) methodology. TURBO DNase (Life Technologies, Grand Island, NY, USA) was used to eliminate genomic DNA, and its measurement was determined by a Qubit® 1.0 fluorometer (Qiagen). The reactions to the synthesis of cDNA were made based on reverse transcriptase using the High-Capacity cDNA Reverse Transcription (ThermoFisher Scientific) kit, and the reactions of qPCR were performed using TaqMan™ Master Mix II (ThermoFisher Scientific) following the manufacturer’s instructions.

Each reaction had a final volume of 10 μL, consisting of: 0.5 μL of TaqMan® Small RNA Assay (20X); 0.66 μL cDNA; 5 μL of TaqMan® Universal PCR Master Mix II (2x) and 3.83 μL of Nuclease free water with the TaqMan® probe (Invitrogen™ ThermoFisher Scientific—KIT q-PCR Assay). The analysis was performed using the 2^-ΔΔCT^. All samples were normalized using endogenous control for glyceraldehyde-3-phosphate dehydrogenase (GAPDH) (Hs04420632_g1)^76,77^. This assay was performed in three individual replicates, conducted on different days.

### Effect of HDPs, MTA, and combination of both on pulp cells’ cytotoxicity and morphology

#### Pulp cell cytotoxicity by MTT

Cell viability was evaluated using the 3-(4,5-dimethylthiazol-2-yl)-2,5-diphenyltetrazolium bromide (MTT) assay, a widely accepted method for assessing cellular metabolic activity. Pulp cells were grown in 96-well plates (1 × 10^4^ cells per well) in 200μL DMEM supplemented medium, previously described, and 10% fetal bovine serum. After 24 h, 16 µg mL^−1^ of the HDPs and MTA extracts were inserted. Plates were incubated at 37 ºC and 5% CO_2_ for 24, 48, and 72 h. After this, the culture medium was removed, 100 μL DMEM supplemented, and 10 μL MTT (5 mg mL^−1^) (Sigma) was added for 4 h, in an incubator at 37ºC and 5% CO_2_. After formazan crystals had been dissolved by DMSO dimethyl sulfoxide (Sigma), optical density was measured by spectrophotometer (Bio-Tek PowerWave HT, EUA), at 570 nm^72^. The test was performed in technical triplicate and three individual replicates, conducted on different days.

#### Pulp cell morphology by scanning electron microscopy (SEM)

Cell morphology was assessed after treatment with HDPs, MTA repair HP, and a combination of both by scanning electron microscope (JSM-7000F Scanning Microscope, JEOL, USA). For this, 18 mm × 18 mm square glass coverslips (Fisher Scientific, Suwanee, GA, USA) were positioned at the base of the 6-well plate (Prolab, São Paulo, SP, Brazil) before cell culture. Then, 2 mL of DMEM culture medium supplemented with 10% fetal bovine serum was inserted, followed by 2 × 10^5^ pulp cells. After 24 h, peptides and MTA extract were inserted, which remained for another 24 h. After this period, the coverslip was fixed in 2.5% glutaraldehyde (Dinâmica, Indaiatuba, São Paulo, Brazil) for 24 h, followed by two washes with sodium cacodylate (Sigma). Subsequently, cells were fixed in osmium tetroxide 1% (Sigma) for 30 min. Later, dehydration in acetone solution (Dinâmica) 30, 50, 70, 95, and 100% was performed. After the coverslip dried, it was metalized and analyzed using a scanning electron microscope (JMST33A Scanning Microscope, JEOL, USA). Images were captured in 300x, 1500x, and 3000 × magnifications to evaluate cell morphology. The test was performed in three individual replicates, conducted on different days.

### Effect of HDPs, MTA, and combination of both on pulp cell migration and proliferation

#### Pulp cell proliferation

Pulp cells were seeded in 96-well plates at a density of 1 × 10^4^ cells per well and cultured for 24 h. Subsequently, the cells were exposed to HDPs, MTA Repair HP extract, and a combination of both. The cell cultures were then incubated at 37ºC and 5% CO_2_ for 24 and 48 h to allow for cellular proliferation. After the specified experimental time points, cell proliferation was assessed using Trypan Blue stain (Sigma). To perform this evaluation, the cells were detached using trypsin (0.025%) and EDTA (0.01%) solution^78^. Following detachment, a 0.4% solution of Trypan Blue stain (Sigma) was added to the cell suspension and incubated for 1 min. The stained cells were then immediately counted using a Neubauer chamber (Brand GmbH, Wertheim, Germany) under a microscope at 400X magnification. The test was performed in technical triplicate and three individual replicates, conducted on different days, of each sample at each experimental time and compared with the initial cell number of the experiment.

#### Pulp cell migration

Pulp cells (2.5 × 10^5^ cells/well) were seeded in technical triplicate in 6-well culture plates (Prolab, Brazil) in supplemented DMEM medium (Gibco). Cultures were maintained until the formation of a confluent monolayer. Next, an artificial wound was reproduced on well surfaces with a plastic micropipette tip. The remaining cells were washed three times, and HDPs and MTA Repair HP extracts were added to cultures with supplemented DMEM with 50 U mL^−1^ penicillin (Gibco) and 50 μg mL^−1^ streptomycin (Gibco). Cultures were incubated and monitored for up to 48 h. Microscopy photographs were taken at 0 h, 24 h, and 48 h for analysis with Image J software (National Institutes of Health, Bethesda, MD, USA). Cells that migrated into the wound were counted, and results were expressed as percentages compared to control^79^.The test was performed in three individual replicates, conducted on different days.

### Effect of HDPs, MTA, and combination of both on odontogenic differentiation and biomineralization

#### Odontogenic differentiation

Pulp cells were plated in triplicate in 24-well plates at a density of 5 × 10^4^ cell/well. After reaching the cellular confluence, cells were treated with supplemented DMEM medium (Sigma), containing osteogenic inducers: 100 nM dexamethasone (Sigma-Aldrich), 10 mM 2-β-glycerol-phosphate (Sigma-Aldrich) and 50 μM ascorbic acid (Sigma-Aldrich)^80^ together with the tested products (HDPs, MTA and the combination of peptides with MTA) for 14 days. The osteogenic medium was used as a positive control. Subsequently, procedures for performing qPCR, alkaline phosphatase activity, phosphate quantification and alizarin red staining were conducted.

#### Analysis of gene expression of biomineralization markers—*Real-time quantitative PCR*

The messenger RNA levels of DSPP, TGF-β, and Glyceraldehyde 3-phosphate dehydrogenase—GAPDH were evaluated after 14 days of cell culture exposed to HDPs and MTA, by StepOnePlus™ Real-Time PCR System (Applied Biosystems, ThermoFisher Scientific, California, USA), under conditions recommended by the manufacturer^76,77^ and previously described. The test was performed in technical triplicate and three individual replicates, conducted on different days.

#### Alkaline phosphatase activity

Alkaline phosphatase enzyme (ALP) activity was determined using the colorimetric method with p-nitrophenyl phosphate (pNPP) as the substrate. The alkaline phosphatase diethanolamine activity kit (Sigma) was employed for this assay. Following a 14-day period of cell exposure to the osteogenic medium, HDPs, and MTA, the cells were rinsed twice with PBS and subsequently incubated with 0.05% Triton X-100 for 20 min at room temperature with shaking. The cell suspension was then transferred to a 1.5 mL tube, vortexed for 20 s, centrifuged at 4 °C and 2500 rpm for 15 min, and placed on ice for 20 min. Aliquots of the resulting cell lysate were mixed with p-nitrophenyl phosphate (pNPP) as the substrate and incubated at 37 °C for 60 min. The enzymatic reaction was terminated by the addition of 5 μL of 1N NaOH, and the absorbance was measured at 405 nm using a spectrophotometer (SpectraMax M2, Molecular Devices, USA). To normalize the alkaline phosphatase activity, the total protein content was determined using the Qubit method^80^. The test was performed in technical triplicate and three individual replicates, conducted on different days.

#### Phosphate quantification

Phosphate concentration in the supernatant of the pulp cell cultures was measured after 14 days of incubation under osteogenic conditions. The Phosphate Colorimetric Assay kit (Sigma-Aldrich, USA) was utilized for this analysis, following the manufacturer's instructions. Phosphate levels were quantified in millimolar (mM) units, by comparing the absorbance values with the standard curve provided in the kit (ranging from 0 to 5 nMol)^81^. The test was performed in technical triplicate and three individual replicates, conducted on different days. This assay allowed for the accurate determination of phosphate concentrations in the cell culture supernatant, providing valuable information about the osteogenic activity of the cells. The experiment was performed in accordance with the recommended protocols, and the results were expressed as mean ± standard deviation.

#### Alizarin red staining

After 14 days, the formation of mineralization nodules was assessed by alizarin red (Sigma) stain, following the manufacturer's instructions. Briefly, cells were fixed in 4% formaldehyde for 15 min, rinsed in PBS, and stained with 40 mM alizarin red staining reagent for 30 min. Cells were washed four times with distilled water, followed by an immediate 15-min rinse with PBS to reduce non-specific dying. The resulting samples were qualitatively analyzed and photographed under an inverted microscope^80^. For quantitative analysis, a 4:1 ratio of 10% acetic acid (Dinâmica) and methanol (Dinâmica) was added to each well to dissolve calcified nodules. Then, the supernatant was read by a spectrophotometer at 405 nm^82^. The test was performed in technical triplicate and three individual replicates, conducted on different days.

### Statistical analyses

The experiments were carried out in technical and biological triplicates, and an antibiofilm activity assay was performed in technical and biological duplicates, as used in previous in vitro studies^10,12,13,18,25,34,35^. Results were submitted to the calculation of the mean and standard deviation for each experiment. Then, normality verification by Kolmogorov Smirnov test and subsequent parametric statistics were performed by analyzing one-way ANOVA and, when necessary, two-way ANOVA. Analyses were considered at a 95% significance level, with a p-value < 0.05. Experiments where p-value was less than 0.01 were indicated in figure legend.

### Ethical approval

This project was approved by the Human Research Ethics Committee of Universidade Católica de Brasília (CEP-UCB CAAE: 7287417000000029). The experiments were performed in accordance with ethics guidelines concerning medical science studies of humans.

### Informed consent

Each patient signed the written informed consent.

## Supplementary Information

Supplementary Information 1.

Supplementary Information 2.

Supplementary Information 3.

Supplementary Information 4.

## Data Availability

The datasets used and analyzed during the current study are available from the corresponding author on reasonable request.
