# Supplementary material for: Host defense peptides combined with MTA extract increase the repair in dental pulp cells: in vitro and ex vivo study
Source: Sci Rep. 2023 Jun 12;13:9531. doi: 10.1038/s41598-023-36748-3 (PMC10261146; doi:10.1038/s41598-023-36748-3)
Supplement: Supplementary file 1 — Supplementary Information 1. [file 41598_2023_36748_MOESM1_ESM.docx]

**SUPPLEMENTARY FIGURE LEGENDS**

Supplementary figure 1 - Mass spectrum obtained by MALDI-ToF of the peptide DJK-6 (1667.62 Da) (A) and IDR1018 (1537.62) (B), with purity greater than 95%.

Supplementary figure 2 - (A) Cell viability after cell exposure to DJK-6 peptide for 24h and (B) for 72h. (C) Cell viability after cell exposure to IDR1018 peptide for 24h and (D) for 72h. (E) Evaluation of the number of cells that migrated into the wound using the DJK-6 peptide, at concentrations from 2 to 64 µg.mL^-1^, within 24 and 48 h. (F) Image of cell migration assay in primary pulp culture, using the scratch method, after contact with DJK-6 peptide, at concentrations from 2 to 64 µg.mL^-^1, at times of 0h, 24h and 48h. Dots denote the presence of a cell. (G) Evaluation of the number of cells that migrated into the wound using the peptide IDR1018 at concentrations from 2 to 64 µg.mL^-1^ within 24h. (H) Image of cell migration assay in primary pulp culture, using the scratch method, after contact with IDR1018 peptide, at concentrations from 2 to 64 µg.mL^-1^, at times of 0h, 24h and 48h. Dots denote the presence of a cell. p<0.05 was represented by (*), compared to the unstimulated pulp cell group. Statistical differences verified by one-way ANOVA test and Tukey’s post test.

Supplementary figure 3 - Mean absorbance of cell viability of young *S. mutans* biofilm in vitro. (A) Analysis of the viability of *S. mutans* biofilm after exposure to HDP DJK-6 for 24h. (B) Analysis of the viability of *S. mutans* biofilm after exposure to HDP IDR1018 for 24h. (C) Mean absorbance of the MTA biomaterial evaluated in conjunction with DJK-6 peptide (D) Mean absorbance of the MTA biomaterial evaluated in conjunction with IDR1018 peptides. Comparative analysis with the MTA biomaterial at a 1:1 concentration. The control was represented by chloramphenicol 10 µg.mL^-1^ and the negative control, only by the *S. mutans* bacterium, in BM2 medium. (*) represents statistical differences between control; (#) statistical difference between the concentration of 4 µg.mL^-1^; (&) statistical difference between the concentration of 8 µg.mL^-1^; (+) statistical difference between the concentration of 16 µg.mL^-1^; (δ) statistical difference between the concentration of 32 µg.mL^-1^; (γ) statistical difference between the concentration of 64 µg.mL^-1^, when p<0.05. All graphs represent means and standard error of the absorbance of three biological replicates in triplicate. Statistical differences verified by one-way ANOVA test and Tukey post-test.
