# Supplementary figures and images for: Host defense peptides combined with MTA extract increase the repair in dental pulp cells: in vitro and ex vivo study
Source: Sci Rep. 2023 Jun 12;13:9531. doi: 10.1038/s41598-023-36748-3 (PMC10261146; doi:10.1038/s41598-023-36748-3)

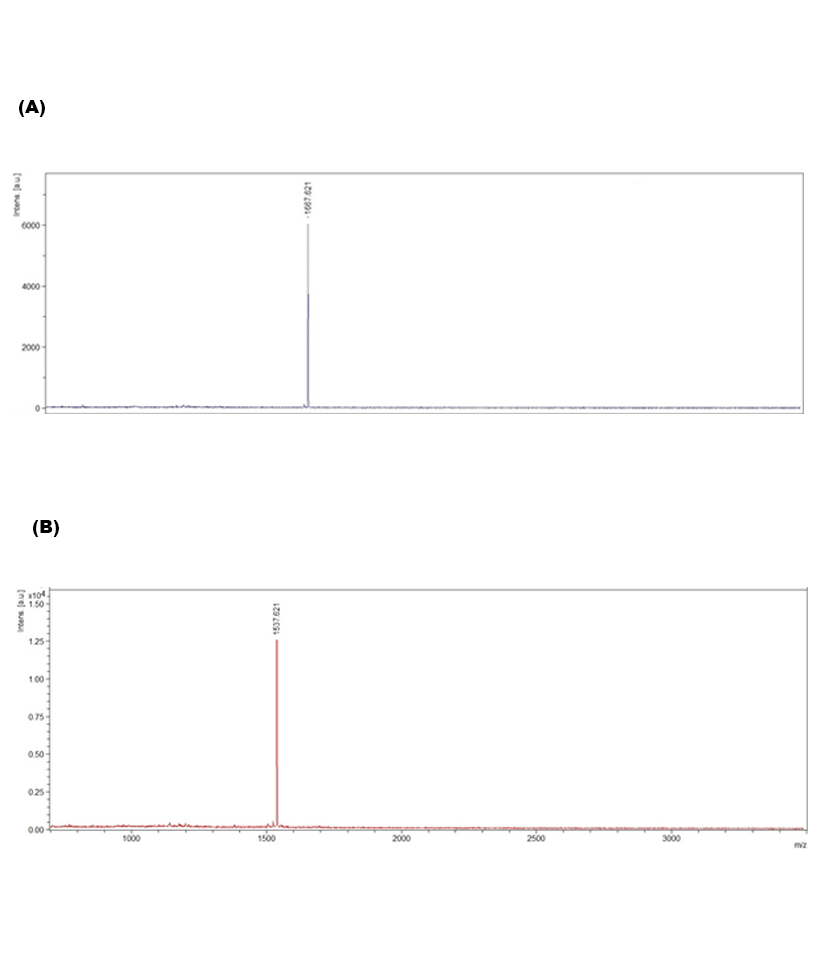

Supplement: Supplementary file 2 — Supplementary Information 2. [file 41598_2023_36748_MOESM2_ESM.tif]

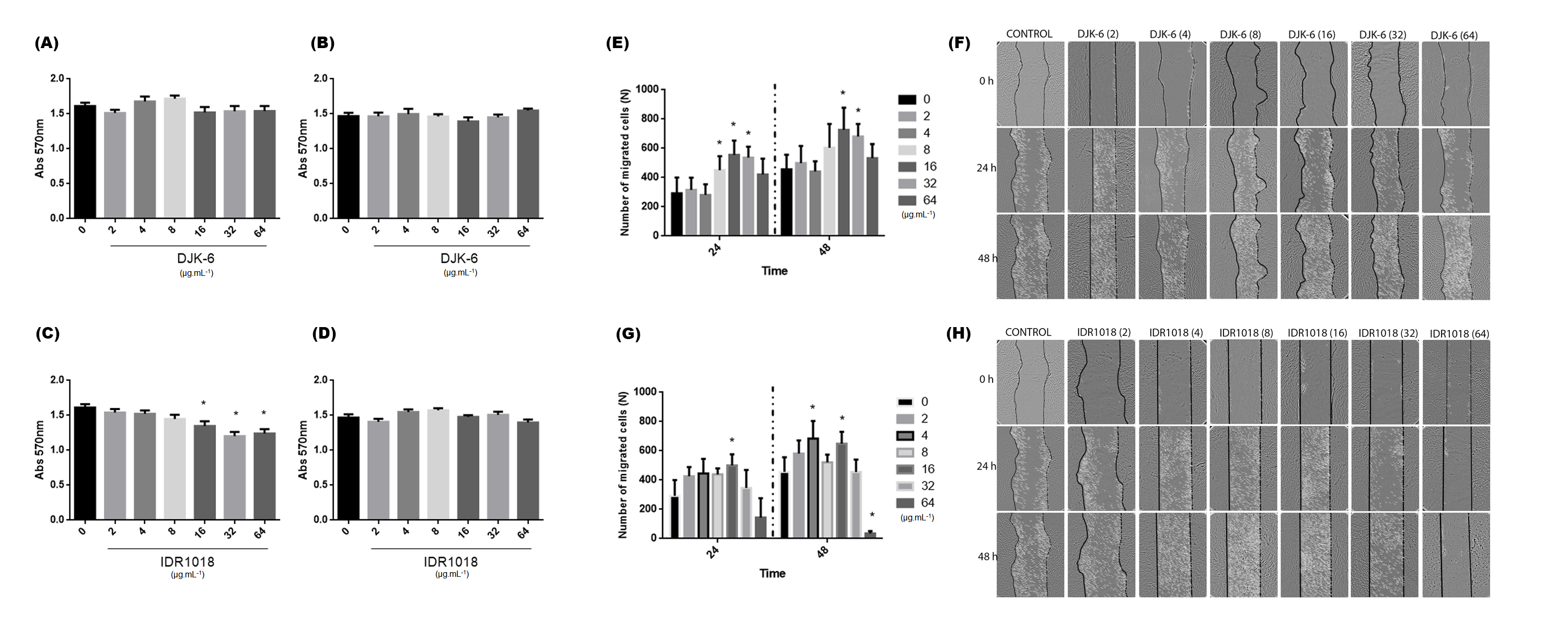

Supplement: Supplementary file 3 — Supplementary Information 3. [file 41598_2023_36748_MOESM3_ESM.tif]

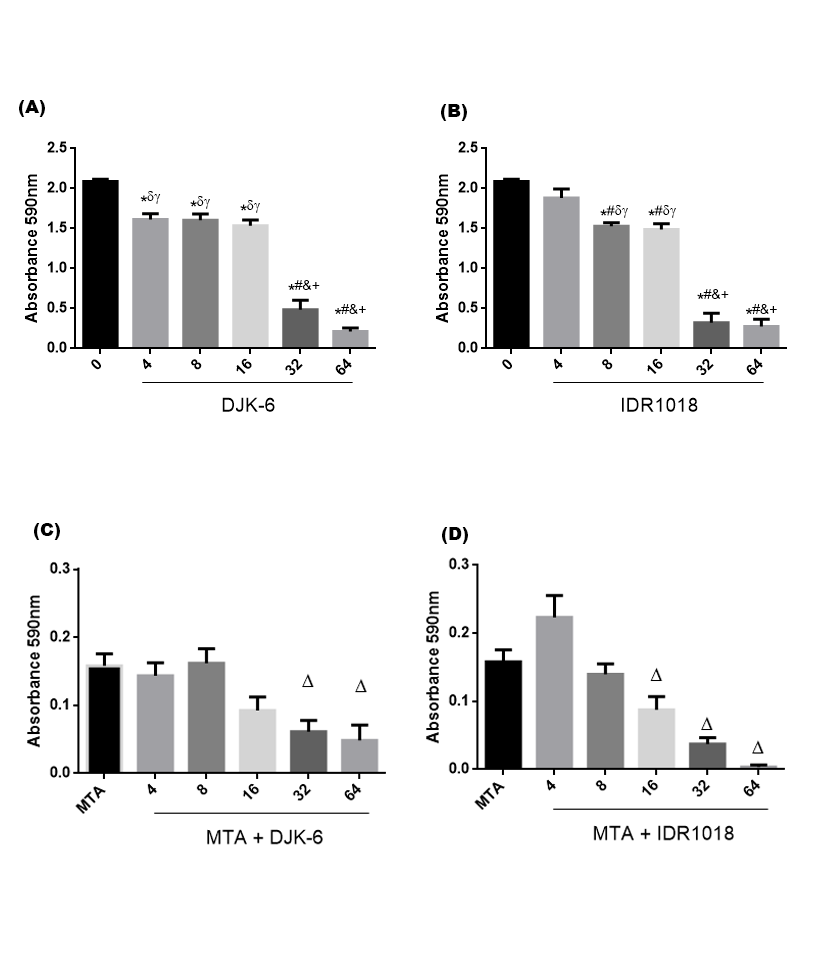

Supplement: Supplementary file 4 — Supplementary Information 4. [file 41598_2023_36748_MOESM4_ESM.tif]
